# Antibacterial, Phytochemical, and Antioxidant Properties of Propolis Extracts Obtained Using Different Solvents Against Multidrug-Resistant Bacteria

**DOI:** 10.3390/pathogens15070749

**Published:** 2026-07-17

**Authors:** Jesús Humberto Reyna-Fuentes, Pablo González-Alanis, Zeferino Blanco-Martínez, Flaviano Benavides-González, Ana Lucía Urbizu-González, María de la Luz Vázquez-Sauceda, Mirelly Venecia Mireles-Villanueva

**Affiliations:** 1 Universidad Tecnológica del Mar Tamaulipas Bicentenario, Soto La Marina-La Pesca, Km. 46, CP. 87678, La Pesca, Soto la Marina 87678, Mexico; jesushumbertoreyna@gmail.com; 2 Facultad de Medicina Veterinaria y Zootecnia, Universidad Autónoma de Tamaulipas, Carretera Victoria-Mante KM 5, Ejido Santa Librada, Ciudad Victoria 87274, Mexico; zblanco@docentes.uat.edu.mx (Z.B.-M.); flbenavides@docentes.uat.edu.mx (F.B.-G.); aurbizu@docentes.uat.edu.mx (A.L.U.-G.); 3 Comisión de Salud Fronteriza México-Estados Unidos, P. del Centenario 10851, Zona Urbana Río Tijuana, Tijuana 22320, Mexico; mirelly2508@gmail.com

**Keywords:** phenolic compounds, flavonoids, biofilm formation, minimum bactericidal concentration, one health

## Abstract

The emergence of multidrug-resistant (MDR) bacteria has increased the need for alternative antimicrobial agents from natural sources. Propolis, a resinous bee product produced by honey bees (*Apis mellifera*) from plant resins, is rich in bioactive compounds with recognized antimicrobial and antioxidant properties. This study evaluated the phytochemical composition, antioxidant capacity, and antibacterial activity of propolis extracts obtained using ethanol, methanol, acetone, and water against clinically relevant MDR bacterial isolates. Total phenolic content was determined using the Folin–Ciocalteu assay, total flavonoid content by the aluminum chloride colorimetric method, and condensed tannin content by the butanol–HCl assay. Antioxidant activity was assessed using the 2,2′-azinobis-(3-ethylbenzothiazoline-6-sulfonic acid) (ABTS), 2,2-diphenyl-1-picrylhydrazyl (DPPH), and ferric reducing antioxidant power (FRAP) assays. Antibacterial activity was evaluated through minimum inhibitory concentration (MIC), minimum bactericidal concentration (MBC), MBC/MIC ratio, biofilm formation inhibition, and bacterial growth inhibition assays against MDR isolates of *Escherichia coli*, *Staphylococcus aureus*, *Pseudomonas aeruginosa*, *Enterococcus faecium*, and *Enterobacter cloacae*. Significant differences among extraction solvents were observed for all phytochemical and antioxidant parameters (*p* < 0.0001). Ethanolic extracts exhibited the highest concentrations of total phenolics, flavonoids, and condensed tannins, as well as the greatest antioxidant activity. MIC values ranged from 3.13 to 25 mg/mL, whereas MBC values ranged from 6.25 to >50 mg/mL depending on bacterial species and extraction solvent. *E. coli* and *E. faecium* were the most susceptible isolates, while *P. aeruginosa* and *E. cloacae* showed greater tolerance. Most MBC/MIC ratios indicated bactericidal activity. Biofilm inhibition was significantly affected by bacterial strain and extraction solvent (*p* < 0.001), with ethanolic extracts producing the highest inhibition percentages. These findings demonstrate that ethanolic propolis extracts exhibit strong antioxidant activity, as well as bacteriostatic, bactericidal, and biofilm-formation-inhibitory effects, supporting their potential as complementary antimicrobial agents within a One Health framework.

## 1. Introduction

The emergence and dissemination of multidrug-resistant (MDR) bacteria present a major challenge to both human and veterinary medicine worldwide [1]. Although the inappropriate and prolonged use of antimicrobial agents has been a major driver of antimicrobial resistance, the emergence and spread of resistant bacteria are multifactorial processes that also involve bacterial genetic adaptation, horizontal gene transfer, and the transmission of resistant microorganisms among humans, animals, and the environment [2]. *Escherichia coli*, *Staphylococcus aureus*, *Pseudomonas aeruginosa*, *Enterococcus faecium*, and *Enterobacter cloacae* are among the most clinically relevant pathogens, frequently associated with opportunistic and chronic infections while exhibiting resistance to multiple antimicrobial classes [3]. As a result, research into natural products with antimicrobial properties has gained considerable attention as an alternative or complementary strategy for combating resistant microorganisms [4].

Propolis is a resinous substance collected by honeybees from plant exudates and subsequently mixed with beeswax and salivary secretions. This bee-derived product is widely recognized for its diverse biological activities, including antimicrobial, antioxidant, anti-inflammatory, antiviral, and immunomodulatory effects [5]. The biological efficacy of propolis is primarily attributed to its complex phytochemical profile, particularly phenolic acids, flavonoids, tannins, terpenoids, and other secondary metabolites [5,6]. However, the chemical composition and bioactivity of propolis can vary substantially according to botanical origin, geographic region, climatic conditions, bee species, and extraction methodology. Among these factors, the extraction solvent plays a decisive role, as it selectively solubilizes distinct groups of bioactive compounds, thereby directly influencing both the phytochemical composition and the biological properties of the resulting extracts [7,8]. Therefore, characterizing propolis from different geographical regions and evaluating the influence of extraction solvents are essential for understanding its therapeutic potential and for optimizing its application as a natural antimicrobial agent [6,9].

Several studies have demonstrated that propolis extracts exhibit inhibitory activity against both Gram-positive and Gram-negative bacteria. However, considerable variability in antibacterial efficacy has been reported, mainly due to differences in phytochemical composition, extraction procedures, and bacterial susceptibility profiles [10]. Beyond its direct antimicrobial activity, propolis is also recognized as an important natural source of antioxidants, including phenolic compounds, flavonoids, and tannins, which help neutralize reactive oxygen species and protect biomolecules from oxidative damage [11].

Since phenolic compounds are widely recognized as major contributors to both the antioxidant and antimicrobial properties of propolis, the simultaneous evaluation of phytochemical composition and antioxidant capacity provides complementary information that helps explain the biological activity of the extracts and the influence of extraction solvents on their therapeutic potential [12].

In addition to inhibiting planktonic bacterial growth, effective antimicrobial candidates should be evaluated for their ability to interfere with biofilm formation, a major virulence factor that contributes to bacterial persistence, chronic infections, and increased tolerance to antimicrobial agents. Because biofilms significantly reduce the effectiveness of conventional treatments, assessing antibiofilm activity together with antibacterial activity provides a more comprehensive evaluation of the therapeutic potential of natural products against MDR pathogens [13,14].

Accordingly, this study aimed to compare the phytochemical composition, antioxidant capacity, antibacterial activity, and antibiofilm potential of propolis extracts obtained using different extraction solvents against five clinically relevant MDR bacterial species (*E. coli*, *S. aureus*, *P. aeruginosa*, *E. faecium*, and *E. cloacae*). Antibacterial activity was assessed using minimum inhibitory concentration (MIC), minimum bactericidal concentration (MBC), MBC/MIC ratio, bacterial growth inhibition, and biofilm inhibition assays, while phytochemical composition and antioxidant activity were determined to better understand the relationships among extraction solvent, chemical composition, and biological activity.

## 2. Materials and Methods

### 2.1. Antimicrobial Activity

#### 2.1.1. Bacterial Strain

The antibacterial activity of propolis extracts was evaluated against five bacterial strains (*E. coli*, *S. aureus*, *P. aeruginosa*, *E. faecium*, and *E. cloacae*) obtained from independent clinical cases. Bacterial isolates were recovered from a laboratory strain collection of clinical origin. For long-term preservation, isolates were stored at −20 °C in Tryptic Soy Broth (TSB; Oxoid Ltd., Basingstoke, UK) supplemented with 20% (*v*/*v*) glycerol. For reactivation, an aliquot of each strain was inoculated into TSB and incubated at 37 °C for 18–24 h under aerobic conditions. Reactivated cultures were streaked onto selective and differential media according to bacterial species. Mannitol Salt Agar (Oxoid Ltd., Basingstoke, UK) was used for *S. aureus*, BD Enterococcosel™ Agar (Becton, Dickinson and Company, Sparks, MD, USA) for *E. faecium*, and MacConkey Agar No. 3 (Oxoid Ltd., Basingstoke, UK) for Gram-negative bacteria, including *E. coli*, *E. cloacae*, and *P. aeruginosa*. Plates were incubated at 37 °C for 18–24 h. Pure cultures were obtained by subculturing representative colonies.

The identity of the clinical bacterial isolates obtained from the laboratory strain collection was confirmed by Gram staining, colony morphology, growth on selective and differential media, and conventional biochemical tests appropriate for each bacterial species, including catalase, coagulase, oxidase, indole production, citrate utilization, triple sugar iron (TSI), urease, and bile esculin hydrolysis, following standard microbiological procedures before antimicrobial susceptibility testing. Antimicrobial susceptibility testing was performed using the disk diffusion (Kirby–Bauer) method on Mueller–Hinton agar (Oxoid Ltd., Basingstoke, UK) according to the Clinical and Laboratory Standards Institute (CLSI) M02 guidelines [15]. Inhibition zone diameters were interpreted according to the CLSI M100 Performance Standards for Antimicrobial Susceptibility Testing, 34th edition (2024) [16]. Results were interpreted qualitatively as susceptible (S) or resistant (R). The isolates were classified as multidrug-resistant (MDR) according to the international criteria proposed by Magiorakos et al. [17], based on antimicrobial susceptibility profiles obtained by the Kirby–Bauer disk diffusion method and interpreted in accordance with CLSI M100 guidelines. The antimicrobial susceptibility profile of each isolate is presented in Table 1.

#### 2.1.2. Propolis Collection and Extraction

Propolis samples were collected from 50 colonies belonging to an experimental apiary comprising approximately 100 honey bee (*A. mellifera* L.) colonies located in Ciudad Victoria, Tamaulipas, Mexico (23°47.57′ N, 99°4.26′ W), using standard plastic mesh traps [18]. The region is characterized by an average annual precipitation of 716 mm, an elevation of 316 m above sea level, and a mean annual temperature of 23.5 °C [19]. Sampling was conducted during the October–December 2025 production season, when bees forage on a wide diversity of flowering plant species; therefore, the collected propolis was of mixed botanical origin (polyfloral). Approximately 200 g of raw propolis was collected from each colony, and all samples were pooled to obtain a composite sample representative of the apiary’s overall phytochemical composition. The samples were kept in darkness and stored at 4 °C until further processing. The propolis mixture was dried in a conventional oven (Lindberg/Blue M, Asheville, NC, USA) at 55 °C for 48 h to reduce moisture content, facilitate homogenization of the sample, and standardize the extraction procedure prior to phytochemical and biological analyses. All samples were subjected to the same drying conditions to ensure consistency among the extraction treatments [20]. Moisture content was determined according to the AOAC method 925.09 [21].

Propolis was extracted using the classical maceration method. Briefly, 20 g of dried propolis was placed in a flask and mixed with 200 mL of 70% (*v*/*v*). Four independent extraction systems were evaluated: 70% ethanol (70:30, ethanol: water, *v*/*v*), 70% methanol (70:30, methanol: water, *v*/*v*), 70% acetone (70:30, acetone: water, *v*/*v*), and distilled water (100%). Each solvent was used separately to prepare the corresponding ethanolic, methanolic, acetone, and aqueous propolis extracts. The mixture was incubated at 35 °C under constant agitation (150 rpm) for 48 h using a shaker (IKA Werke GmbH & Co. KG, Staufen, Germany). After extraction, the mixture was first filtered through Whatman No. 1 filter paper (Cytiva, Marlborough, MA, USA) to remove insoluble residues, then passed through a 0.22 µm membrane filter (Merck Millipore, Burlington, MA, USA) to obtain a clarified extract [22]. The extract was stored in amber glass containers at room temperature and protected from light until analysis.

#### 2.1.3. Determination of Minimal Inhibitory Concentration (MIC)

Antibacterial activity of the propolis extracts was evaluated using the broth microdilution method to determine the minimum inhibitory concentration (MIC) against *E. coli*, *S. aureus*, *P. aeruginosa*, *E. faecium*, and *E. cloacae*, CLSI M100 [16]. Assays were performed in sterile flat-bottom 96-well polystyrene microplates with a final volume of 200 µL per well. Bacterial inocula were prepared from fresh cultures grown on blood agar plates (Oxoid Ltd., Basingstoke, UK) at 37 °C for 18–24 h. Subsequently, 3–5 well-isolated colonies were suspended in sterile Mueller–Hinton broth (MHB) and adjusted to the turbidity of a 0.5 McFarland standard (approximately 1 × 10^8^ CFU/mL) by visual comparison. The suspensions were then diluted in MHB to obtain a final inoculum concentration of approximately 5 × 10^5^ CFU/mL per well.

Serial two-fold dilutions of each propolis extract were prepared in MHB to obtain concentrations expressed in mg/mL. Wells containing only MHB served as sterility controls, whereas wells containing a bacterial inoculum without extract served as growth controls. In addition, solvent-specific controls containing the corresponding extraction solvent (70% ethanol, 70% methanol, 70% acetone, or distilled water) at the same final concentration used in the respective extract dilutions were included to verify that the observed antibacterial activity was attributable to the propolis extracts rather than to the extraction solvents. *Escherichia coli* ATCC 25922 was included as a quality control strain.

Microplates were incubated aerobically at 37 °C for 24 h. The MIC was defined as the lowest concentration of the extract that resulted in a complete absence of visible bacterial growth compared with the growth control wells. Bacterial growth inhibition was additionally quantified by measuring optical density at 630 nm using a microplate reader (iMark™ Microplate Absorbance Reader, model 11293, Bio-Rad Laboratories, Hercules, CA, USA). All assays were performed in triplicate.

#### 2.1.4. Determination of Minimum Bactericidal Concentration (MBC)

The minimum bactericidal concentration (MBC) was determined according to standard microbiological procedures. Following MIC determination, 10 µL aliquots from wells exhibiting complete growth inhibition were subcultured onto Mueller–Hinton agar plates and incubated aerobically at 37 °C for 24 h. The MBC was defined as the lowest concentration of propolis extract that produced a ≥99.9% reduction in viable bacterial cells relative to the initial inoculum. The absence of visible colony growth after incubation was considered indicative of bactericidal activity [23]. All assays were conducted in triplicate.

#### 2.1.5. Determination of the MBC/MIC Ratio

The antibacterial mode of action of propolis extracts was further evaluated by calculating the ratio between the minimum bactericidal concentration (MBC) and the minimum inhibitory concentration (MIC). The MBC/MIC ratio was obtained by dividing the MBC by the corresponding MIC value for each bacterial strain. According to accepted microbiological criteria, extracts were classified as bactericidal when the MBC/MIC ratio was ≤4 and as bacteriostatic when it was >4 [24]. This approach enabled discrimination between the growth-inhibitory and bacterial-killing effects of the evaluated propolis extracts.

(1) $$Ratio=\frac{MBC}{MIC}$$

where MBC is the minimum bactericidal concentration, and MIC is the minimum inhibitory concentration.

#### 2.1.6. Biofilm Formation Inhibition Assay

The ability of propolis extracts to inhibit biofilm formation was evaluated using the crystal violet microtiter plate assay as previously described by [25]. Prior to biofilm evaluation, sub-inhibitory concentrations of each propolis extract were established based on the previously determined minimum inhibitory concentration (MIC) values. Concentrations corresponding to one-half (1/2 MIC) and one-quarter (1/4 MIC) of the MIC were evaluated for each extract–bacterium combination. Bacterial growth at these concentrations was assessed under the same incubation conditions as the biofilm assay by measuring optical density at 630 nm after 24 h. Optical density values were statistically compared with the untreated growth control, and the highest concentration that did not significantly affect planktonic bacterial growth (*p* > 0.05) was selected for subsequent biofilm inhibition experiments.

Bacterial suspensions were adjusted to approximately 1 × 10^8^ CFU/mL (0.5 McFarland standard) and diluted 1:100 in Tryptic Soy Broth (TSB) supplemented with 1% glucose. Aliquots of 100 µL of bacterial suspension and 100 µL of the selected sub-inhibitory concentration of each propolis extract were added to sterile flat-bottom 96-well polystyrene microplates. Wells containing bacterial suspension without extract served as positive controls, whereas wells containing sterile medium alone served as negative controls. Plates were incubated at 37 °C for 24 h under static conditions to allow biofilm formation.

After incubation, planktonic cells were removed, and wells were gently washed three times with sterile phosphate-buffered saline (PBS). The attached biofilms were fixed with methanol for 15 min and stained with 0.1% crystal violet solution for 15 min. Excess stain was removed by washing with distilled water, and the plates were air-dried. Bound crystal violet was solubilized using 95% ethanol, and absorbance was measured at 570 nm using a microplate reader (iMark™ Microplate Absorbance Reader, model 11293, Bio-Rad Laboratories, Hercules, CA, USA). The percentage of biofilm inhibition was calculated using the following equation:

(2) $$Biofilm\;Inhibition=\frac{OD\;control-OD\;treatment}{OD\;control}\;\times 100$$

### 2.2. Determination of Secondary Compound

#### 2.2.1. Flavonoid Content

The total flavonoid content was determined as previously described [26]. In brief, 10 µL of the extract was mixed with 1.5 mL of 95% ethanol (Ibi Scientific, no. 15720, Dubuque, IA, USA), 2.7 µL of 10% aluminum chloride (Sigma-Aldrich, St. Louis, MO, USA), 2.7 µL of 1 M potassium acetate (Sigma-Aldrich, St. Louis, MO, USA), and 102.7 µL of distilled water, and incubated for 40 min at room temperature. The samples were read at 415 nm (iMark™ Microplate Absorbance Reader, model 11293, Bio-Rad Laboratories, Hercules, CA, USA) and quantified using a standard curve prepared with quercetin (Sigma-Aldrich, St. Louis, MO, USA), described by the equation y = 0.0215x + 0.0121 (R^2^ = 0.9975). The concentration of total flavonoids is expressed as mg quercetin equivalents per g of dry matter (mg QE/g).

#### 2.2.2. Phenolic Content

The total phenolic content was determined using the Folin–Ciocalteu method [27]. In brief, 2 µL of the extracts was deposited into a 96-well plate and mixed with 25 µL of Folin–Ciocalteu reagent (Sigma-Aldrich, St. Louis, MO, USA). The samples were homogenized and incubated at room temperature for 5 min. Then, 125 µL of 10 mM NaCO_3_ (Sigma-Aldrich, St. Louis, MO, USA) and 49 µL of distilled water were added, and the mixture was incubated at room temperature for an additional 5 min. The samples were read at 750 nm (iMark™ Microplate Absorbance Reader, model 11293, Bio-Rad Laboratories, Hercules, CA, USA) and quantified with gallic acid as the standard. The calibration curve is described by the equation y = 0.037x + 0.1237 (R^2^ = 0.9961). The concentration of total phenols is expressed as mg gallic acid equivalents per gram of dry matter (mg GAE/g).

#### 2.2.3. Condensed Tannin Content

Condensed tannin content was determined using the HCl-butanol assay described by [28]. Briefly, 3 mL of HCl-butanol reagent was added to 0.5 mL of each extract, followed by 0.1 mL of ferric reagent (HCl-NH_4_Fe(SO_4_)_2_). The mixtures were transferred into screw-cap test tubes (13 × 100 mm), tightly sealed, and incubated in a water bath at 100 °C for 1 h. After cooling to room temperature, absorbance was measured at 460 nm using a UV–Vis spectrophotometer (Genesys 10S UV–Vis, Thermo Fisher Scientific, Madison, WI, USA). Tannin concentrations were calculated using a catechin standard curve (y = 0.0082x + 0.0254; R^2^ = 0.9968) and expressed as milligrams of catechin equivalents per gram of sample (mg CE/g). 

### 2.3. Antioxidant Activity Assays

#### 2.3.1. ABTS Radical Cation Scavenging Assay

The ABTS radical cation-scavenging activity was determined according to the method of [29]. The ABTS•^+^ radical was generated by mixing 7 mM ABTS with 2.45 mM potassium persulfate and allowing the mixture to stand in the dark at room temperature for 12–16 h. Before analysis, the ABTS•^+^ solution was diluted with ethanol to obtain an absorbance of 0.70 ± 0.02 at 734 nm. The reaction mixture in a 96-well microplate consisted of 1.25 µL of propolis extract and 125 µL of ABTS•^+^ working solution. The mixture was incubated in the dark at room temperature for 6 min. Absorbance was measured at 734 nm using a microplate reader (iMark™ Microplate Absorbance Reader, model 11293, Bio-Rad Laboratories, Hercules, CA, USA). A control, solvent blank, and sample blank were included to correct for background absorbance. Results were expressed as µmol Trolox equivalents per gram of dry extract (µmol TE/g) using a Trolox calibration curve described by the equation y = 0.1132x + 1.904 (R^2^ = 0.9984). All assays were performed in triplicate.

#### 2.3.2. DPPH Radical Scavenging Assay

The free radical scavenging activity of the propolis extracts (acetone, aqueous, ethanolic and methanolic) was evaluated using the DPPH assay, following the method of [30]. A stock solution of DPPH (0.6 mM) was prepared in methanol and diluted to obtain a working solution with an absorbance of 1.1 ± 0.02 at 517 nm. The reaction mixture in a 96-well microplate consisted of 7.5 µL of propolis extract and 150 µL of DPPH working solution. The mixture was incubated in the dark at room temperature for 30 min. Absorbance was measured at 517 nm using a microplate reader (iMark™ Microplate Absorbance Reader, model 11293, Bio-Rad Laboratories, Hercules, CA, USA). A control (DPPH solution without sample), a solvent blank, and a sample blank (extract without DPPH) were included to correct for background interference. Antioxidant activity was expressed as Trolox-equivalent antioxidant capacity (TEAC), reported as µmol Trolox equivalents per gram of dry extract (µmol TE/g), based on a calibration curve constructed using Trolox and described by the equation y = 0.0914x + 2.184 (R^2^ = 0.9951). All assays were performed in triplicate.

#### 2.3.3. Ferric Reducing Antioxidant Power (FRAP) Assay

The FRAP method is based on the reduction of ferric iron (Fe^3+^) in the FRAP reagent to ferrous iron (Fe^2+^) in the presence of antioxidants [31]. For sample analysis, 900 µL of freshly prepared FRAP reagent, 30 µL of propolis extract, and 120 µL of distilled water were mixed. The FRAP reagent was prepared by mixing 25 mL of acetate buffer (acetic acid–sodium acetate, pH 3.6), 2.5 mL of 10 mM TPTZ solution prepared in 40 mM HCl, and 2.5 mL of 20 mM FeCl_3_ solution. The formation of a blue-colored ferrous–TPTZ complex, proportional to the sample’s reducing capacity, was measured at 593 nm using a microplate reader (iMark™ Microplate Absorbance Reader, model 11293, Bio-Rad Laboratories, Hercules, CA, USA). A control sample was included for each measurement, and its absorbance was used to correct the background. Antioxidant activity was quantified using a Trolox standard curve (100–1000 µmol/L) prepared in ethanol and described by the equation y = 0.0056x + 0.0372 (R^2^ = 0.9973). Results were expressed as µmol Trolox equivalents per gram of dry extract (µmol TE/g). All assays were performed in triplicate.

### 2.4. Experimental Design

Antibacterial activity was evaluated using a broth microdilution assay in sterile 96-well microplates. The experimental factors included bacterial species (*E. coli*, *S. aureus*, *P. aeruginosa*, *E. faecium*, and *E. cloacae*), extraction solvent (70% ethanol, 70% methanol, 70% acetone, and distilled water), and propolis extract concentration (mg/mL, via a two-fold serial dilution). Each well was considered an experimental unit. The following controls were included in each plate: (i) sterility control (broth only), (ii) growth control (broth plus bacterial inoculum), (iii) solvent-specific controls consisting of culture broth containing the corresponding extraction solvent at the same final concentration used in the respective test wells, and (iv) the quality control strain *Escherichia coli* ATCC 25922. The MIC was defined as the lowest concentration of propolis extract showing no visible bacterial growth compared with the growth control. For the biofilm inhibition assay, sub-inhibitory concentrations (1/2 MIC and 1/4 MIC) were initially evaluated for each extract–bacterium combination. The highest concentration that did not significantly affect planktonic bacterial growth was subsequently selected and used for biofilm formation assays. All experiments were performed in triplicate, each conducted in three independent experiments.

### 2.5. Statistical Analysis

Data were analyzed using parametric or non-parametric statistical procedures depending on the assumptions of normality and homogeneity of variances. The antibacterial activity of propolis extracts, expressed as minimum inhibitory concentration (MIC), minimum bactericidal concentration (MBC), MBC/MIC ratio, bacterial growth (optical density, OD_630_), and verification of sub-inhibitory concentrations prior to the biofilm inhibition assay, was evaluated using the Kruskal–Wallis test. When significant differences were detected, pairwise comparisons were performed using Dunn’s test with Bonferroni correction or the Steel–Dwass method for multiple comparisons. The relationship between propolis extract concentration and bacterial growth inhibition (OD_630_) was assessed using Spearman’s rank correlation coefficient (ρ) and Kendall’s tau correlation coefficient (τ). Biofilm inhibition data were analyzed using a two-way analysis of variance (ANOVA), with bacterial strain and extraction solvent (70% ethanol, 70% methanol, 70% acetone, and aqueous) as fixed factors. When significant effects were detected, means were separated using Tukey’s honestly significant difference (HSD) test.

For secondary compounds (total phenolics, flavonoids, and condensed tannins) and antioxidant activity (ABTS, DPPH, and FRAP), data were analyzed using one-way analysis of variance (ANOVA), followed by Tukey’s HSD test for multiple comparisons when assumptions were met. Correlations among phytochemical compounds and antioxidant activity assays were evaluated using Pearson’s correlation coefficient (r), and the results were visualized using a correlation heatmap. All statistical analyses were performed using JMP Pro 17 [32]. Results were considered statistically significant at *p* < 0.05.

## 3. Results

### 3.1. Antibacterial Activity

The MIC assay showed that all propolis extracts inhibited the growth of the MDR bacterial isolates evaluated (Table 2). Among the bacterial strains, *E. coli* and *E. faecium* exhibited the highest susceptibility to the ethanolic extract, with the lowest MIC values (3.13 mg/mL). In contrast, *P. aeruginosa* and *E. cloacae* generally required higher concentrations across all propolis extracts, indicating lower overall susceptibility.

The minimum bactericidal concentration (MBC) assay confirmed the antibacterial activity of propolis extracts obtained using different solvents against all MDR bacterial isolates evaluated (Table 3). Overall, MBC values followed a susceptibility pattern similar to that observed in the MIC assay, with ethanolic and methanolic extracts exhibiting the strongest bactericidal activity. Among the bacterial strains evaluated, *Escherichia coli* and *Enterococcus faecium* showed the lowest MBC values, particularly in ethanolic and methanolic extracts (6.25 mg/mL), indicating high susceptibility to propolis’s bactericidal effects. In contrast, *Pseudomonas aeruginosa* and *Enterobacter cloacae* required higher extract concentrations to achieve complete bacterial killing. MBC values ranged from 25 to >50 mg/mL for *P. aeruginosa* and from 50 to >50 mg/mL *for E. cloacae*, depending on the extraction solvent.

The MBC/MIC ratios for propolis extracts obtained with different solvents are shown in Table 4. Most extract–bacteria combinations exhibited MBC/MIC ratios ranging from 2.0 to 4.0, indicating predominantly bactericidal activity according to accepted microbiological criteria [23]. The lowest ratios (2) were observed for *Escherichia coli*, *Staphylococcus aureus*, *Enterococcus faecium*, and the reference strain *E. coli* ATCC 25922, demonstrating high susceptibility to the bactericidal effects of propolis extracts.

In contrast, *Enterobacter cloacae* exhibited a higher MBC/MIC ratio (8) when treated with the ethanolic extract, indicating reduced susceptibility to propolis’s bactericidal activity. Furthermore, the bactericidal endpoint was not reached at the highest concentration tested (>50 mg/mL) for some extract–bacteria combinations, particularly *Pseudomonas aeruginosa* exposed to acetone and aqueous extracts, and *Enterobacter cloacae* exposed to methanolic and aqueous extracts. These results suggest that Gram-negative bacteria generally exhibited greater tolerance to the bactericidal effects of propolis extracts than Gram-positive bacteria.

Prior to evaluating biofilm inhibition, it was necessary to verify that the selected sub-inhibitory concentrations did not significantly impair planktonic bacterial growth, thereby ensuring that any subsequent reduction in biofilm formation could be attributed to antibiofilm activity rather than to bacterial growth inhibition. Significant differences among treatments were observed for *E. coli* exposed to the ethanol (χ^2^ = 11.66, df = 2, *p* = 0.0029), methanol (χ^2^ = 11.42, df = 2, *p* = 0.0033), and acetone (χ^2^ = 11.50, df = 2, *p* = 0.0032) extracts, whereas no significant differences were detected for the aqueous extract (χ^2^ = 0.44, df = 2, *p* = 0.8010) (Table 5). Similar patterns were observed for *S. aureus* and *E. faecium*, in which the ethanol, methanol, and acetone extracts significantly reduced planktonic bacterial growth at 1/2 MIC (*p* < 0.05), while bacterial growth at 1/4 MIC did not differ significantly from the untreated growth control. Consequently, 1/4 MIC was selected for these extract–bacterium combinations.

In contrast, no significant differences among treatments were observed for *P. aeruginosa* or *E. cloacae* regardless of the extraction solvent (*p* > 0.05). Therefore, 1/2 MIC was selected as the highest concentration that did not significantly affect planktonic bacterial growth. For the aqueous extract, significant reductions in bacterial growth at 1/2 MIC were observed only for *S. aureus* and *E. faecium*, whereas no significant differences were detected between the untreated control and 1/4 MIC, leading to the selection of 1/4 MIC for these two species. Conversely, the aqueous extract did not significantly affect planktonic growth of *E. coli*, *P. aeruginosa*, or *E. cloacae* at either sub-inhibitory concentration. Therefore, 1/2 MIC was selected for the subsequent biofilm inhibition assay (Table 5).

Biofilm inhibition evaluated using the selected sub-inhibitory concentrations (sub-MIC) was significantly affected by bacterial strain (F = 38.72, *p* < 0.0001), extraction solvent (F = 146.58, *p* < 0.0001), and the bacterial strain × solvent interaction (F = 4.36, *p* = 0.0004). The ethanolic extract consistently exhibited the highest biofilm inhibition percentages across all MDR bacterial isolates evaluated, followed by methanolic, acetone, and aqueous extracts (Table 6).

Among the bacterial strains, *S. aureus* and *E. faecium* were the most susceptible to the anti-biofilm effects of propolis extracts. Treatment with the ethanolic extract resulted in biofilm inhibition values of 69.8 ± 5.1% and 65.6 ± 4.9%, respectively. In contrast, *P. aeruginosa* exhibited the lowest inhibition percentages across all extraction solvents, ranging from 18.7 ± 3.1% to 43.5 ± 4.7%, indicating greater tolerance to propolis treatment. Intermediate inhibition levels were observed for *E. coli* and *E. cloacae*.

**Figure 1:**
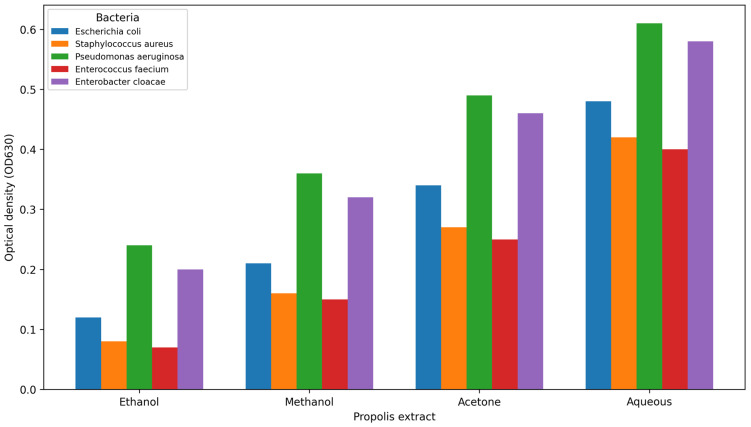
Average bacterial growth (OD_630_) of MDR bacterial isolates exposed to propolis extracts prepared with different solvents.

The results revealed significant differences among treatments (Kruskal–Wallis test, *p* < 0.05), indicating that at least one solvent extract exhibited antibacterial activity distinct from the others (Figure 1). Overall, the ethanolic extract showed the lowest OD_630_ values across all MDR bacterial isolates evaluated, indicating greater bacterial growth inhibition than the methanolic, acetone, and aqueous extracts. Gram-positive bacteria, particularly *Staphylococcus aureus* and *Enterococcus faecium*, exhibited the highest susceptibility to the ethanolic extract, with OD_630_ values below 0.10. In contrast, *Pseudomonas aeruginosa* and *Enterobacter cloacae* exhibited higher OD_630_ values across all solvent extracts, indicating lower susceptibility to propolis treatment. Methanolic extracts also demonstrated antibacterial activity, although with reduced effectiveness compared with ethanolic extracts. Acetone extract and aqueous extract exhibited the highest OD_630_ values overall, indicating lower antibacterial activity against the evaluated bacterial strains. The quality control strain, *E. coli* ATCC 25922, showed high susceptibility to all solvent extracts, particularly the ethanolic extract.

Subsequently, Dunn’s multiple comparisons test with Bonferroni correction was performed to identify which solvent extracts exhibited significant differences in antibacterial activity (Table 7). Significant differences were observed between ethanolic and aqueous extracts (*p* < 0.001) and between ethanolic and acetone extracts (*p* = 0.003). These findings confirm the superior antibacterial activity of the ethanolic propolis extract compared with the other solvent extracts evaluated.

### 3.2. Dose-Dependent Bacterial Growth Inhibition During the MIC Assay

To complement the MIC determination, optical density (OD_630_) values obtained during the broth microdilution assay were analyzed to quantitatively evaluate the effects of propolis concentration, extraction solvent, and bacterial species on bacterial growth inhibition.

The Kruskal–Wallis test revealed a highly significant effect of propolis extract concentration on bacterial growth inhibition (χ^2^ = 116.66, df = 9, *p* < 0.0001). Median OD_630_ values progressively decreased with increasing extract concentration, confirming a clear dose–response relationship between propolis concentration and antibacterial activity. Pairwise Steel–Dwass comparisons grouped concentrations into distinct categories (Table 8). Lower concentrations (97.66–390.62 µg/mL) exhibited the highest median OD_630_ values, indicating reduced antibacterial activity, whereas intermediate concentrations showed a gradual reduction in bacterial growth. Concentrations ≥ 6250 µg/mL clustered in the lowest OD_630_ groups, indicating maximal inhibitory activity against the evaluated bacterial isolates.

Significant differences in antibacterial activity were observed among the solvent extracts evaluated (χ^2^ = 53.38, df = 3, *p* < 0.0001). Ethanolic extracts consistently exhibited the lowest OD_630_ values, indicating greater bacterial growth inhibition than methanolic, acetone, and aqueous extracts. In contrast, the aqueous extract exhibited the highest OD_630_ values overall, indicating reduced antibacterial activity against the MDR bacterial strains evaluated. Pairwise Steel–Dwass comparisons revealed significant differences between the ethanolic and aqueous extracts, as well as between the ethanolic and acetone extracts (*p* < 0.0001). However, no significant difference was observed between ethanolic and methanolic extracts (*p* = 0.1683), suggesting similar antibacterial efficacy across these solvent systems.

**Figure 2:**
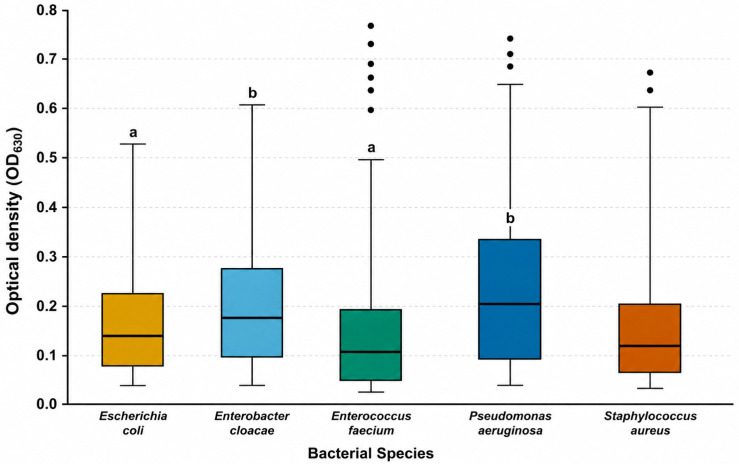
Variation in bacterial growth inhibition measured as optical density (OD_630_) among MDR bacterial species exposed to propolis extracts. Boxes sharing the same lowercase letter are not significantly different, whereas boxes with different lowercase letters differ significantly according to the Steel–Dwass multiple comparison test (*p* < 0.05).

Bacterial species differed significantly in their optical density (OD_630_) in response to propolis extracts (χ^2^ = 24.65, df = 4, *p* < 0.0001). *E. faecium* and *S. aureus* were the most susceptible, exhibiting lower OD_630_ values, whereas *P. aeruginosa* and *E. cloacae* showed the highest OD_630_ values, indicating greater tolerance of these bacteria to propolis treatment (Figure 2). Steel–Dwass comparisons confirmed significant differences among bacterial species, particularly between *P. aeruginosa* and *E. faecium* (*p* = 0.0011) and between *E. cloacae* and Gram-positive bacteria (*p* < 0.05).

The nonparametric correlation analysis revealed a strong, highly significant negative relationship between propolis extract concentration and bacterial growth, measured as optical density (OD_630_). At the global level, the analysis yielded a Spearman’s rank correlation coefficient of ρ = −0.765 (*p* < 0.0001) and a Kendall’s tau correlation coefficient of τ = −0.584 (*p* < 0.0001), confirming a monotonic decrease in bacterial growth with increasing propolis concentration. When analyzed across MDR bacterial species, all isolates exhibited concentration-dependent inhibition by propolis extracts. *P. aeruginosa* consistently exhibited higher OD_630_ values across the evaluated concentration range, indicating greater bacterial tolerance to treatment, whereas *E. faecium* and *S. aureus* showed lower OD_630_ values, reflecting greater susceptibility to the antibacterial activity of propolis extracts.

### 3.3. Phytochemical Composition and Antioxidant Activity

Significant differences were identified among extraction solvents for all evaluated phytochemical and antioxidant variables (*p* < 0.0001). Ethanolic extracts consistently exhibited the highest concentrations of bioactive compounds and antioxidant activity, while methanolic and acetone extracts showed progressively lower values (Table 9).

Total phenolic content varied significantly among extraction solvents (F = 86.86, *p* < 0.0001). Ethanolic extracts exhibited the highest phenolic concentration (357.11 ± 21.41 mg GAE/g), followed by methanolic extracts (290.71 ± 22.83 mg GAE/g), while acetone and aqueous extracts showed substantially lower values.

Total flavonoid content was also significantly affected by extraction solvent (F = 38.09, *p* = 0.0004). Ethanolic extracts exhibited the highest flavonoid concentration (42.07 ± 5.95 mg QE/g), followed by methanolic extracts (31.66 ± 8.42 mg QE/g), whereas acetone and aqueous extracts contained considerably lower concentrations (<21 mg QE/g). Condensed tannin content also differed significantly among extraction solvents (F = 17.09, *p* = 0.0033). Ethanolic extracts contained the highest tannin concentration (17.61 ± 2.29 mg CE/g), while methanolic and acetone extracts exhibited intermediate values, and the aqueous extract showed the lowest tannin content.

Antioxidant activity differed significantly among extraction solvents in all assays (DPPH: F = 168.68, *p* < 0.0001; ABTS: F = 314.46, *p* < 0.0001; FRAP: F = 141.50, *p* < 0.0001). In the DPPH assay, ethanolic extracts exhibited the highest radical-scavenging activity (428.68 ± 37.16 µmol TE/g), whereas aqueous extracts showed the lowest values (218 ± 17.39 µmol TE/g). A similar solvent-dependent trend was observed in the ABTS assay, with antioxidant capacity ranging from 1485.35 ± 116.92 µmol TE/g in ethanolic extracts to 784.00 ± 80.52 µmol TE/g in aqueous extracts. Likewise, FRAP values were significantly higher in ethanolic extracts (690.45 ± 74.47 µmol TE/g) than in aqueous extracts (300.47 ± 22.72 µmol TE/g), while methanolic and acetone extracts consistently exhibited intermediate antioxidant activities.

### 3.4. Correlation Analysis

**Figure 3:**
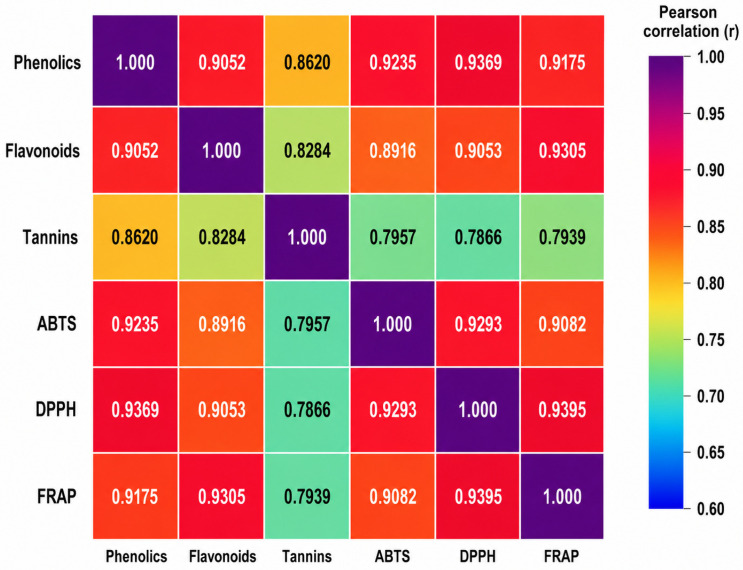
Pearson correlation heatmap among phytochemical compounds and antioxidant activity assays of propolis extracts. Color intensity represents the strength of correlation coefficients (r).

The correlation analysis identified strong positive associations between phytochemicals and antioxidant activity in propolis extracts (Figure 3). Total phenolic content exhibited high and significant correlations with ABTS (r = 0.9235, *p* < 0.0001), DPPH (r = 0.9369, *p* < 0.0001), and FRAP (r = 0.9175, *p* < 0.0001), indicating that phenolic compounds were strongly associated with the antioxidant capacity of the evaluated extracts. Total flavonoid content also showed strong positive correlations with ABTS (r = 0.8916, *p* < 0.0001), DPPH (r = 0.9053, *p* < 0.0001), and FRAP (r = 0.9305, *p* < 0.0001). These results indicate that flavonoids were important contributors to the radical-scavenging and ferric-reducing activities of propolis extracts.

Condensed tannins showed significant positive correlations with antioxidant assays, though these associations were weaker than those observed for phenolics and flavonoids. Correlation coefficients between tannins and antioxidant activity were 0.7957 for ABTS (*p* = 0.0103), 0.7866 for DPPH (*p* = 0.0119), and 0.7939 for FRAP (*p* = 0.0106).

Strong correlations were observed among antioxidant assays. ABTS exhibited positive correlations with DPPH (r = 0.9293, *p* < 0.0001) and FRAP (r = 0.9082, *p* < 0.0001), and DPPH and FRAP were also highly correlated (r = 0.9395, *p* < 0.0001).

## 4. Discussion

The present study demonstrated that propolis extracts obtained using different solvents possess considerable antibacterial activity against clinically relevant MDR bacterial isolates, including *E. coli*, *S. aureus*, *P. aeruginosa*, *E. faecium*, and *E. cloacae*. Overall, ethanolic extracts consistently exhibited the greatest antibacterial efficacy, characterized by lower MIC values, reduced optical density (OD_630_), and stronger inhibition of bacterial proliferation compared with methanolic, acetone, and aqueous extracts. These findings reinforce the growing evidence that propolis is a promising natural alternative for controlling resistant bacterial pathogens and support its potential application as a complementary antimicrobial agent in veterinary and biomedical contexts [33,34,35].

The superior antibacterial performance observed in ethanolic extracts is likely attributable to ethanol’s greater extraction efficiency for a wide range of bioactive constituents present in propolis [36,37]. Owing to its intermediate polarity, ethanol efficiently extracts phenolic acids, flavonoids, aromatic acids, esters, terpenoids, and other secondary metabolites, yielding extracts that are chemically richer than those from highly polar solvents such as water or from solvents with different extraction affinities, such as acetone. Consequently, solvent polarity strongly influences the phytochemical composition and biological activity of propolis extracts. The relatively homogeneous antibacterial activity observed for the aqueous extract across most bacterial species may also be explained by its extraction profile. Because water primarily solubilizes highly polar constituents, it generally extracts lower concentrations of flavonoids, aromatic acids, terpenoids, and other less polar bioactive metabolites associated with antibacterial activity. The similar MIC values observed for the aqueous extract across most bacterial species may be attributed to its extraction profile. Because water primarily extracts highly polar constituents, the resulting extract likely contains lower concentrations and a less diverse composition of antibacterial metabolites than the organic extracts. Consequently, its antibacterial activity was more consistent across the evaluated bacterial species than that of the organic extracts [38].

Similar findings have been reported for propolis from different geographical regions, where ethanolic extracts consistently exhibited greater antibacterial activity than aqueous or acetone extracts against both Gram-positive and Gram-negative bacteria [39]. The antimicrobial activity of propolis cannot be attributed exclusively to phenolic compounds. Instead, it is likely the result of synergistic interactions among multiple classes of bioactive constituents, whose combined effects enhance antibacterial efficacy through multitarget mechanisms. Flavonoids increase membrane permeability, disrupt proton gradients, inhibit ATP synthesis, and interfere with DNA gyrase and topoisomerase activities, whereas phenolic acids destabilize bacterial membranes and induce intracellular oxidative stress. Condensed tannins contribute through protein precipitation, metal ion chelation, and inhibition of extracellular enzymes, while terpenoids further enhance antibacterial activity by altering membrane integrity. Collectively, these complementary mechanisms likely explain the broad-spectrum antibacterial activity of propolis and reduce the likelihood that bacteria will develop resistance through a single cellular target or mechanism of action [12,40,41].

Gram-positive bacteria, particularly *Staphylococcus aureus* and *Enterococcus faecium*, were more susceptible to propolis extracts than Gram-negative bacteria, whereas *Pseudomonas aeruginosa* and *Enterobacter cloacae* exhibited greater tolerance [42,43]. This difference is mainly attributed to the outer membrane of Gram-negative bacteria, which restricts the penetration of hydrophobic bioactive compounds, including many constituents of propolis [44]. In contrast, the absence of this barrier in Gram-positive bacteria facilitates interactions between phytochemicals and cellular targets, resulting in lower MIC values, as consistently reported for propolis from diverse geographical origins [10,40,45]. This pattern was further supported by the MBC results, which showed that ethanolic and methanolic extracts required lower concentrations to achieve bactericidal activity, particularly against *E. coli*, *S. aureus*, and *E. faecium*, whereas *P. aeruginosa* and *E. cloacae* remained the least susceptible species. Their greater tolerance is consistent with intrinsic resistance mechanisms, including reduced membrane permeability, multidrug efflux pumps, enzymatic detoxification systems, and adaptive stress responses that limit the intracellular accumulation of antimicrobial compounds [46,47]. Likewise, most extract–bacteria combinations exhibited MBC/MIC ratios ≤ 4, indicating predominantly bactericidal activity according to accepted microbiological criteria [48]. Similar findings have been reported for propolis and other phenolic-rich natural products, whose bactericidal or bacteriostatic effects depend on bacterial susceptibility, phytochemical composition, and extract concentration [49].

In addition to inhibiting planktonic bacterial growth, propolis extracts markedly reduced biofilm formation, with the ethanolic extract exhibiting the greatest activity, particularly against *S. aureus* and *E. faecium*. Biofilms are major virulence factors because they protect bacteria from antimicrobial agents and host immune defenses, promote horizontal gene transfer, and contribute to chronic and recurrent infections [50]. Because the biofilm assay in the present study was performed at the MIC, the observed reduction in biofilm biomass cannot be exclusively attributed to specific antibiofilm mechanisms. Instead, it likely reflects the combined effects of bacterial growth inhibition and reduced biofilm development. Nevertheless, previous studies performed under sub-MIC conditions have demonstrated that propolis and its bioactive constituents interfere with several stages of biofilm development, including bacterial adhesion, extracellular polymeric substance (EPS) production, quorum-sensing signaling, and biofilm maturation [51,52]. Therefore, future studies evaluating sub-MICs will be necessary to determine whether the propolis extracts investigated here directly modulate these antibiofilm mechanisms independently of their antibacterial activity.

The enhanced antibiofilm activity observed for the ethanolic extract is likely related to its greater recovery of bioactive metabolites. However, this activity cannot be attributed exclusively to phenolic compounds, since flavonoids, terpenoids, aromatic acids, esters, and other secondary metabolites may act through multiple complementary mechanisms that impair bacterial adhesion, EPS synthesis, and quorum-sensing signaling. The combined action of these phytochemicals may weaken biofilm architecture, increase bacterial susceptibility to antimicrobial agents, and reduce the likelihood of resistance development by acting on a single cellular target or mechanism of action [53,54,55]. Consequently, propolis represents a promising multifunctional natural product that may complement existing antimicrobial therapies, particularly against biofilm-associated infections, within a One Health framework. Furthermore, its generally favorable safety profile and multitarget mechanisms of action support continued investigation of propolis as an adjunctive strategy for controlling MDR infections.

The dose-dependent reduction in bacterial growth further supports the antimicrobial potential of propolis extracts. Increasing extract concentrations progressively reduced OD_630_ values, demonstrating a clear concentration-dependent antibacterial effect. This response is likely associated with the greater availability of bioactive metabolites that can simultaneously target bacterial membranes, intracellular enzymes, nucleic acids, and other essential metabolic processes [56]. Similar dose-dependent effects have been reported for propolis from different geographical regions, where increasing extract concentrations consistently reduced bacterial viability and biofilm formation [37,57].

Phytochemical analysis revealed that ethanolic extracts contained significantly higher concentrations of total phenolics, flavonoids, and condensed tannins than methanolic, acetone, and aqueous extracts [58,59,60]. This pattern is consistent with the superior antibacterial activity observed for ethanolic extracts. Although phenolic compounds are recognized as major contributors to the biological activity of propolis, other constituents, including terpenoids, aromatic acids, esters, and additional secondary metabolites, are also likely to contribute through complementary mechanisms [61,62,63,64].

The higher antioxidant capacity of ethanolic extracts, demonstrated by the DPPH, ABTS, and FRAP assays, was positively correlated with their phenolic and flavonoid contents. The consistent superiority of ethanolic extracts across all three assays indicates that ethanol efficiently recovered compounds capable of acting through multiple antioxidant pathways [65,66,67]. The antioxidant activity of propolis may also enhance its antimicrobial potential by reducing oxidative damage, modulating cellular redox balance, and potentiating the biological activity of other bioactive metabolites, thereby contributing to the overall therapeutic potential of the extracts [68].

In addition, condensed tannins showed positive correlations with antioxidant activity and may further contribute to antimicrobial efficacy through protein precipitation, metal ion chelation, inhibition of extracellular enzymes, and interference with bacterial adhesion [69,70]. Finally, the strong positive correlations among the ABTS, DPPH, and FRAP assays demonstrate the consistency of the antioxidant profiles of the evaluated propolis extracts despite the different reaction mechanisms of each method. Similar relationships have been reported for propolis from diverse botanical and geographical origins, supporting the reliability of these assays and reinforcing the importance of phenolic-rich propolis as a valuable natural source of antioxidant and antimicrobial compounds [71,72,73,74].

Despite the promising findings, this study has some limitations. Antibiofilm activity was evaluated only at the MIC; the intrinsic biofilm-forming capacity of the clinical isolates was not characterized, and the maximum concentration tested (50 mg/mL) prevented determination of higher MIC and MBC values for some extract–bacterium combinations. Future studies should identify the specific bioactive compounds responsible for the observed effects, evaluate antibiofilm activity at sub-MICs, and confirm the safety and efficacy of propolis through toxicity and in vivo studies.

## 5. Conclusions

The present study demonstrated that the extraction solvent markedly influences the phytochemical composition and biological activity of propolis. Among the extraction systems evaluated, the ethanolic extract consistently exhibited the greatest antibacterial and antibiofilm activities against clinically relevant MDR bacterial isolates, underscoring the importance of solvent selection in maximizing propolis’s biological potential. Overall, these findings support the potential of propolis as a promising natural source of bioactive compounds that may complement existing antimicrobial strategies within a One Health framework. However, further studies are needed to identify the specific compounds responsible for these biological effects, evaluate antibiofilm activity at sub-MICs, characterize the intrinsic biofilm-forming capacity of the bacterial isolates, and assess toxicity and in vivo efficacy before clinical or veterinary applications can be considered.

## Figures and Tables

**Table 1:** Antimicrobial susceptibility profiles of multidrug-resistant (MDR) bacterial isolates used in the antibacterial evaluation of propolis extracts.

Antimicrobial Class	Antimicrobial Subclass	Antimicrobial Agent	*E. coli*	*S. aureus*	*P. aeruginosa*	*E. faecium*	*E. cloacae*
β-lactams	Penicillins	Ampicillin	R	–	R	R	R
β-lactams	Penicillins	Amoxicillin/ clavulanic acid	R	S	R	–	R
β-lactams	Cephalosporins	Cefpodoxime	R	–	R	–	R
β-lactams	Cephalosporins	Ceftazidime	R	–	R	–	R
β-lactams	Carbapenems	Imipenem	S	S	S	–	S
Fluoroquinolones	–	Ciprofloxacin	R	R	R	R	R
Fluoroquinolones	–	Enrofloxacin	R	R	R	S	R
Tetracyclines	–	Doxycycline	R	S	–	R	R
Phenicols	–	Chloramphenicol	R	S	–	S	S
Sulfonamides	–	Trimethoprim/ sulfamethoxazole	R	R	–	–	R
Macrolides	–	Erythromycin	–	R	–	R	–
Aminoglycosides	–	Gentamicin	R	R	R	–	R
Lincosamides	–	Clindamycin	–	R	–	–	–
Tetracyclines	–	Tetracycline	–	R	–	R	–

Antimicrobial susceptibility testing was performed using the Kirby–Bauer disk diffusion method according to CLSI M02 guidelines. Interpretation of susceptibility was based on the CLSI M100 Performance Standards for Antimicrobial Susceptibility Testing, 34th edition (2024). S = susceptible; R = resistant; – = not tested or not applicable.

**Table 2:** Minimum inhibitory concentration (MIC, mg/mL) of propolis extracts against MDR bacterial isolates.

	Propolis Extract			
MDR Strain	Ethanol	Methanol	Acetone	Aqueous Extract
*Escherichia coli*	3.13	6.25	12.5	12.5
*Staphylococcus aureus*	6.25	3.13	6.25	12.5
*Pseudomonas aeruginosa*	6.25	12.5	25	12.5
*Enterococcus faecium*	3.13	3.13	6.25	12.5
*Enterobacter cloacae*	6.25	12.5	12.5	12.5
*E. coli* ATCC 25922	3.13	3.13	3.13	6.25

**Table 3:** Minimum bactericidal concentration (MBC, mg/mL) of propolis extracts against MDR bacterial isolates.

	Propolis Extract			
MDR Strain	Ethanol	Methanol	Acetone	Aqueous Extract
*Escherichia coli*	6.25	12.50	25	25
*Staphylococcus aureus*	12.50	6.25	12.50	25
*Pseudomonas aeruginosa*	25	50	>50	>50
*Enterococcus faecium*	6.25	6.25	12.50	25
*Enterobacter cloacae*	50	>50	50	>50
*E. coli* ATCC 25922	6.25	6.25	6.25	12.50

**Table 4:** MBC/MIC ratio of propolis extracts obtained using different solvents against MDR bacterial isolates.

Bacterial Strain	Ethanol	Methanol	Acetone	Aqueous Extract
*Escherichia coli*	2	2	2	4
*Staphylococcus aureus*	2	2	4	4
*Pseudomonas aeruginosa*	4	4	ND	ND
*Enterococcus faecium*	2	2	2	2
*Enterobacter cloacae*	8	ND	4	ND
*E. coli* ATCC 25922	2	2	2	2

ND = bactericidal endpoint not reached at the highest concentration tested (>50 mg/mL).

**Table 5:** Selection of sub-inhibitory concentrations (sub-MIC) used for the biofilm inhibition assay based on planktonic bacterial growth.

Bacterial Strain	Propolis Extract	Growth Control (OD_630_)	1/2 MIC (OD_630_)	1/4 MIC (OD_630_)	Selected Concentration
*Escherichia coli*	Ethanol	0.646 (0.635–0.656) ^a^	0.451 (0.442–0.459) ^b^	0.639 (0.625–0.649) ^a^	1/4 MIC
	Methanol	0.658 (0.650–0.666) ^a^	0.464 (0.455–0.472) ^b^	0.649 (0.643–0.650) ^a^	1/4 MIC
	Acetone	0.654 (0.648–0.662) ^a^	0.548 (0.541–0.557) ^b^	0.643 (0.640–0.645) ^a^	1/4 MIC
	Aqueous	0.673 (0.662–0.681) ^a^	0.664 (0.656–0.670) ^a^	0.667 (0.648–0.673) ^a^	1/2 MIC
*Staphylococcus aureus*	Ethanol	0.555 (0.552–0.561) ^a^	0.389 (0.382–0.397) ^b^	0.554 (0.551–0.558) ^a^	1/4 MIC
	Methanol	0.565 (0.559–0.573) ^a^	0.398 (0.390–0.405) ^b^	0.562 (0.550–0.573) ^a^	1/4 MIC
	Acetone	0.571 (0.565–0.577) ^a^	0.453 (0.448–0.462) ^b^	0.575 (0.572–0.577) ^a^	1/4 MIC
	Aqueous	0.584 (0.578–0.592) ^a^	0.489 (0.481–0.494) ^b^	0.581 (0.575–0.592) ^a^	1/4 MIC
*Pseudomonas aeruginosa*	Ethanol	0.798 (0.793–0.807) ^a^	0.789 (0.783–0.798) ^a^	0.796 (0.796–0.803) ^a^	1/2 MIC
	Methanol	0.817 (0.810–0.825) ^a^	0.812 (0.805–0.820) ^a^	0.821 (0.809–0.832) ^a^	1/2 MIC
	Acetone	0.827 (0.820–0.835) ^a^	0.823 (0.816–0.830) ^a^	0.835 (0.818–0.847) ^a^	1/2 MIC
	Aqueous	0.840 (0.833–0.847) ^a^	0.834 (0.826–0.842) ^a^	0.839 (0.824–0.842) ^a^	1/2 MIC
*Enterococcus faecium*	Ethanol	0.499 (0.492–0.505) ^a^	0.345 (0.338–0.352) ^b^	0.496 (0.488–0.502) ^a^	1/4 MIC
	Methanol	0.518 (0.514–0.522) ^a^	0.361 (0.356–0.368) ^b^	0.520 (0.514–0.521) ^a^	1/4 MIC
	Acetone	0.523 (0.518–0.530) ^a^	0.409 (0.402–0.416) ^b^	0.526 (0.518–0.534) ^a^	1/4 MIC
	Aqueous	0.535 (0.529–0.540) ^a^	0.447 (0.442–0.454) ^b^	0.534 (0.528–0.536) ^a^	1/4 MIC
*Enterobacter cloacae*	Ethanol	0.724 (0.718–0.731) ^a^	0.715 (0.708–0.722) ^a^	0.734 (0.710–0.744) ^a^	1/2 MIC
	Methanol	0.737 (0.731–0.744) ^a^	0.731 (0.724–0.738) ^a^	0.740 (0.730–0.752) ^a^	1/2 MIC
	Acetone	0.721 (0.715–0.728) ^a^	0.716 (0.708–0.722) ^a^	0.716 (0.700–0.734) ^a^	1/2 MIC
	Aqueous	0.736 (0.731–0.744) ^a^	0.730 (0.725–0.737) ^a^	0.734 (0.730–0.749) ^a^	1/2 MIC

Values are presented as the median (interquartile range, IQR). Different superscript letters within the same row indicate significant differences among treatments (Growth control, 1/2 MIC, and 1/4 MIC) according to the Kruskal–Wallis test followed by the Steel–Dwass multiple comparison test (*p* < 0.05). The selected sub-inhibitory concentration is the highest concentration that did not significantly affect planktonic bacterial growth relative to the untreated growth control.

**Table 6:** Biofilm inhibition (%) of propolis extracts evaluated at the selected sub-inhibitory concentrations (sub-MIC) against MDR bacterial isolates.

Bacterial Strain	Ethanol	Methanol	Acetone	Aqueous Extract
*Escherichia coli*	58.9 ± 5.6 ^a^	52.7 ± 5.1 ^b^	39.8 ± 4.4 ^c^	28.4 ± 3.9 ^d^
*Staphylococcus aureus*	69.8 ± 5.1 ^a^	64.3 ± 4.8 ^ab^	48.6 ± 4.5 ^c^	35.1 ± 4.2 ^d^
*Pseudomonas aeruginosa*	43.5 ± 4.7 ^a^	37.6 ± 4.1 ^b^	25.2 ± 3.6 ^c^	18.7 ± 3.1 ^d^
*Enterococcus faecium*	65.6 ± 4.9 ^a^	60.8 ± 4.6 ^ab^	45.2 ± 4.3 ^c^	33.9 ± 3.8 ^d^
*Enterobacter cloacae*	49.7 ± 5.0 ^a^	42.4 ± 4.3 ^b^	30.3 ± 3.7 ^c^	22.1 ± 3.4 ^d^

Values are expressed as mean ± standard deviation. Different superscript letters within rows indicate significant differences among extraction solvents according to Tukey’s honestly significant difference (HSD) test (*p* < 0.05).

**Table 7:** Pairwise comparisons of antibacterial activity among propolis extracts obtained using Dunn’s test with Bonferroni correction.

Solvent Comparison	*p*-Value
Ethanol–Methanol	0.041
Ethanol–Acetone	0.003
Ethanol–Aqueous extract	<0.001
Methanol–Acetone	0.072
Methanol–Aqueous extract	0.015
Acetone–Aqueous extract	0.084

**Table 8:** Dose-dependent inhibition of bacterial growth by increasing propolis extract concentrations (µg/mL).

Propolis Concentration (µg/mL)	Median OD_630_	IQR
97.66	0.60 ^a^	0.19
195.31	0.56 ^a^	0.18
390.62	0.51 ^b^	0.16
781.25	0.45 ^c^	0.14
1562.5	0.39 ^d^	0.12
3125	0.31 ^e^	0.1
6250	0.24 ^f^	0.08
12,500	0.18 ^f^	0.06
25,000	0.11 ^f^	0.05
50,000	0.06 ^f^	0.03

Groups with different letters differ significantly according to the Steel–Dwass test (*p* < 0.05). Values are expressed as median (IQR).

**Table 9:** Phytochemical composition and antioxidant activity of propolis extracts obtained using different extraction solvents.

Propolis Extract						
Solvent	Total Phenolic Content (mg GAE/g)	Flavonoid Content (mg QE/g)	Condensed Tannins (mg CE/g)	ABTS (µmol TE/g)	DPPH (µmol TE/g)	FRAP (µmol TE/g)
Acetone	210.07 ± 18.60 ^c^	20.83 ± 3.38 ^c^	9.23 ± 1.70 ^b^	845.63 ± 67.34 ^c^	263.72 ± 11.62 ^c^	382.15 ± 19.58 ^c^
Aqueous	202.21 ± 15.61 ^c^	18.36 ± 2.19 ^c^	4.67 ± 0.88 ^d^	784 ± 80.52 ^c^	218 ± 17.39 ^d^	300.47 ± 22.72 ^d^
Ethanol	357.11 ± 21.41 ^a^	42.07 ± 5.95 ^a^	17.61 ± 2.29 ^a^	1485.35 ± 116.92 ^a^	428.68 ± 37.16 ^a^	690.45 ± 74.47 ^a^
Methanol	290.71 ± 22.83 ^b^	31.66 ± 8.42 ^b^	11.24 ± 3.11 ^b^	1264.84 ± 158.96 ^b^	361.43 ± 43.55 ^b^	574.33 ± 62.25 ^b^
*p* value	<0.0001	0.0004	0.0033	<0.0001	<0.0001	<0.0001

Values are expressed as mean ± standard deviation. Different superscript letters in the columns indicate significant differences according to Tukey’s multiple comparisons test (*p* < 0.05). GAE: gallic acid equivalents; QE: quercetin equivalents; CE: catechin equivalents; TE: Trolox equivalents.

## Data Availability

All data generated or analyzed during this study are included in this article. Additional information supporting the findings of this study can be obtained from the corresponding author upon reasonable request.

## Abbreviations

The following abbreviations are used in this manuscript:

MIC	Minimum concentration inhibitory
*v*/*v*	Volume/volume
MDR	multidrug-resistant
GAE	Gallic acid equivalent
QE	Quercetin equivalent
FRAP	Ferric Reducing Antioxidant Power
°C	degrees Celsius
mL	milliliter
rpm	revolutions per minute
µm	Micrometer
h	Hour
MHB	Mueller-Hinton
µL	Microliter
CFU	colony-forming units
nm	nanometer
min	minute
mg	milligram
TPTZ	2,4,6-Tris(2-pyridyl)-s-triazine
mM	Millimolar
NaCO_3_	Sodium Carbonate
Fe^+3^	Ferric iron
Fe^+2^	Ferrous
pH	Hydrogen potential
HCl	Hydrochloric acid
No.	Number
μmol	micromole
OD	Optical density
ANOVA	Analysis of variance
SAS	Statistical Analysis System
IQR	Interquartile rang
df	Degree freedom
g	grams
TE	Trolox equivalent
TFC	Total flavonoid content
TPC	Total phenolic content
CLSI	Clinical and Laboratory Standards Institute
AOAC	Association of Official Analytical Communities
